# The impact of mental health disorders in pregnancy on obstetric and neonatal outcomes: A narrative review

**DOI:** 10.1016/j.bbih.2026.101343

**Published:** 2026-08-29

**Authors:** Tiziano Prodi, Melissa Rosa, Marta Spinoni, Claudio Singh Solorzano, Maria Grazia Di Benedetto, Marco Andrea Riva, Bernardo Dell’Osso, Annamaria Cattaneo

**Affiliations:** a Department of Pharmacological and Biomolecular Sciences, University of Milan, Milan, Italy; b Department of Psychiatry, Department of Biomedical and Clinical Sciences, University of Milan, Milan, Italy; c Biological Psychiatry Unit, IRCCS Istituto Centro San Giovanni di Dio Fatebenefratelli, Brescia, Italy; d “Aldo Ravelli" Center for Neurotechnology and Brain Therapeutic, University of Milan, Milan, Italy; e Department of Psychiatry and Behavioral Sciences, Bipolar Disorders Clinic, Stanford University, CA, USA; f Centro per Lo Studio Dei Meccanismi Molecolari Alla Base Delle Patologie Neuro-psico-geriatriche”, University of Milan, Milan, Italy

**Keywords:** Perinatal psychiatry, Pregnancy, Mental disorders, Maternal health, Obstetric outcomes, Neonatal outcomes

## Abstract

Perinatal psychiatry has gained increasing attention as a public health priority, given the high prevalence and burden of mental health conditions during pregnancy and the postpartum period. While research has predominantly focused on depressive disorders, growing evidence suggests that a broader range of psychiatric conditions may significantly influence obstetric and neonatal outcomes. Unlike previous works primarily centered on perinatal depression, this narrative review provides an integrated and transdiagnostic overview of perinatal psychiatric disorders – including mood, anxiety, psychotic, obsessive-compulsive, trauma-related disorders, and borderline personality disorder – and their association with adverse maternal, obstetric, and neonatal outcomes. Across diagnostic categories, psychiatric disorders during pregnancy are consistently associated with increased risks of hypertensive disorders, preterm birth, altered fetal growth, operative delivery, and neonatal complications. However, findings remain heterogeneous, largely derived from observational studies, and often limited by variability in diagnostic assessment and outcome definitions. Shared pathophysiological pathways– including hypothalamic-pituitary-adrenal axis dysregulation, inflammatory activation, hormonal fluctuations, and psychosocial stressors – may partially explain the convergence of different psychiatric disorders on similar perinatal outcomes. Taken together, these findings highlight the need to move beyond a depression-centered framework in perinatal psychiatry. Early identification, individualized risk–benefit assessment, and integrated multidisciplinary care may contribute to improved maternal and offspring outcomes. Future research should prioritize longitudinal designs and standardized outcome measures to disentangle disorder-specific and shared mechanisms.

## 1 Introduction

Attention to perinatal psychiatry – defined as the prevention, diagnosis and treatment of mental health conditions during pregnancy and the first year postpartum – has increased in recent years (Casanova Dias and Jones, 2024). In particular, the perinatal period represents a time of heightened vulnerability, during which mental health disorders may emerge or worsen through a bidirectional relationship: pregnancy can influence the trajectory of pre-existing mental health conditions, whereas psychiatric disorders may adversely affect both maternal and fetal wellbeing (Casanova Dias and Jones, 2024). Notably, together with substance use disorders, perinatal mental health conditions are among the leading causes of morbidity and mortality during this period (Deichen Hansen et al., 2023). Beyond their direct psychiatric burden, perinatal mental health conditions are associated with an increased risk of adverse obstetric and neonatal outcomes, including preterm birth and low birth weight, thereby underscoring their relevance for comprehensive perinatal care (Grigoriadis et al., 2018). However, despite their substantial burden, perinatal mental health disorders remain underdiagnosed and undertreated, resulting in detrimental consequences for women, their offspring, and the broader family system (Deichen Hansen et al., 2023; Turchioe et al., 2024; Zimmermann et al., 2024).

To date, research in perinatal psychiatry has focused predominantly on depressive disorders, leading to the development of validated screening tools, clinical guidelines, and preventive interventions. While this focus has significantly improved the identification and management of perinatal depression, evidence regarding other psychiatric disorders in the perinatal context remains limited and fragmented (Vigod et al., 2025). The Canadian Network for Mood and Anxiety Treatments (CANMAT) has explicitly called for further investigation into perinatal aspects of psychiatric conditions other than depression, which continue to be underrepresented in the literature (Vigod et al., 2025). Available studies are often heterogeneous, primarily observational in design, and characterized by variability in diagnostic assessment and outcome definitions, limiting the comparability of findings (Aujla et al., 2024; Grigoriadis et al., 2018). Moreover, although the association between perinatal mental health conditions and adverse obstetric, fetal, and neonatal outcomes has been acknowledged by the American College of Obstetricians and Gynecologists (ACOG), this recognition has not yet been systemically integrated across diagnostic categories ("Screening and Diagnosis of Mental Health Conditions during Pregnancy and Postpartum", 2023). A clearer and more comprehensive understanding of how diverse mental health conditions could influence pregnancy, delivery, and neonatal outcomes is therefore essential to inform clinical decision-making, optimize perinatal care, and guide future research directions.

Against this background, an integrated overview of perinatal psychiatric disorders and their association with obstetric and neonatal outcomes is warranted. In the following sections we synthesize the existing literature focusing on the most recent evidence regarding the obstetric and neonatal outcomes associated with perinatal mental health conditions focusing on major psychiatric disorders with established clinical relevance during the perinatal period, selected based on their prevalence among women of reproductive age and their documented vulnerability during pregnancy and the postpartum period (Howard and Khalifeh, 2020; Vigod et al., 2025). Specifically, these conditions include mood, anxiety, psychotic, obsessive-compulsive, and trauma-related disorders. Borderline personality disorder was additionally included given its relatively high prevalence in clinical perinatal populations and its documented impact on both maternal and offspring outcomes (Prasad et al., 2022). The aim of this narrative review is to provide a structured and clinically meaningful overview of perinatal psychiatric disorders and their association with obstetric and neonatal outcomes. By integrating evidence across diagnostic categories, we summarize current knowledge regarding adverse outcomes affecting women during pregnancy and delivery, as well as their offspring, and highlight clinically relevant implications and key gaps to inform future research.

## 2 Literature search strategy

This narrative review was informed by a structured, non-systematic search of the PubMed database, initially conducted in January 2026 and subsequently updated during manuscript preparation. Three authors contributed to the literature search according to a shared search framework, with separate searches conducted for each psychiatric condition considered in the review rather than using a single comprehensive search string. The searches combined Medical Subject Headings (MeSH) and free-text terms related to the perinatal period (e.g., “pregnancy”, “perinatal”, “antenatal”, “prenatal”), the specific psychiatric condition under investigation (e.g., “depression”, “anxiety”, “schizophrenia”, “psychosis”, “bipolar disorder”, “obsessive-compulsive disorder”, “OCD”, “post-traumatic stress disorder”, “PTSD”, “trauma”, “borderline personality disorder”, “BPD”), and obstetric and neonatal outcomes (e.g., “pregnancy outcome”, “obstetric*”, “birth outcome”, “preterm”, “low birth weight”, “cesarean”, “neonatal*”). Terms were combined as appropriate using the Boolean operators AND and OR. Only English-language publications were considered. Systematic reviews, meta-analyses, clinical guidelines, and recent or methodologically informative original studies were prioritized, while additional relevant publications were identified through manual screening of the reference lists of selected articles. Given the broad and multifaceted nature of the topic and the heterogeneity of the available evidence across psychiatric conditions and obstetric and neonatal outcomes, a narrative approach was considered the most appropriate to provide an integrated, clinically oriented, transdiagnostic synthesis of the current literature.

## 3 Psychiatric disorders in pregnancy and Obstetric–Neonatal outcomes

International epidemiological studies estimate that approximately one in five women experiences an anxiety disorder during the perinatal period (Fawcett et al., 2019). Consistently, the Australian Institute of Health and Welfare (AIHW) reports a prevalence of 10–20%, identifying anxiety disorders as the most common mental health conditions during this period (Australian Institute of Health and Welfare [AIHW], 2024).

Perinatal psychiatric disorders encompass a broad spectrum of conditions, including depressive, trauma-related, bipolar, schizophrenia-spectrum, obsessive-compulsive, and personality disorders, all of which may substantially affect maternal and offspring health. In the following sections, we summarize available evidence on the association between major psychiatric disorders and obstetric and neonatal outcomes. For each diagnostic category, we focus on the most relevant maternal, obstetric, and neonatal complications reported in the literature.

### 3.1 Depressive disorders

Depressive disorders are among the most common mental health conditions affecting women of reproductive age and represent a leading cause of maternal morbidity and disability during pregnancy and the childbearing years (Liao et al., 2025). Antenatal depression substantially affects maternal quality of life and constitutes a major source of disease burden in both developed and developing countries (Dadi et al., 2020). According to this, the AIHW reported that perinatal depression occurs in the 10% of the mothers in high-income countries, whereas the prevalence in the developing countries reaches the 20%, as report in the World Health Organization (WHO) report (AIHW, 2024; World Health Organization, nd).

Approximately 7% of women worldwide experience depression, and the risk nearly doubles during pregnancy, with a prevalence of MDD during pregnancy estimated at approximately 12–13%, while up to 30–37% of women report clinically relevant depressive symptoms at some point during gestation (Howard and Khalifeh, 2020; Kessler et al., 2003; Lee et al., 2007; Marcus and Heringhausen, 2009).

Several risk factors for antenatal depression have been identified and may be broadly categorized into biological, psychological, social, and obstetric domains. These include hormonal fluctuations, chronic stress, anxiety symptoms, low social support, interpersonal difficulties, unintended or unplanned pregnancy, exposure to violence, and a personal or family history of depression (Bromet et al., 2011; Goldstein et al., 2014; Goodman, 2007; Soares and Zitek, 2008; Sundström Poromaa et al., 2017).

Multiple studies and meta-analyses have investigated the association between antenatal depression and adverse pregnancy outcomes, although findings remain partly inconsistent (Alder et al., 2007; Bonari et al., 2004; Kramer, 1987; López et al., 2024; Zhou et al., 2024). Some evidence suggests that depression during pregnancy is significantly associated with preterm birth, low birth weight, and intrauterine growth restriction, whereas other studies have reported no direct association (Accortt et al., 2015; Araujo et al., 2010; Byrn and Penckofer, 2013; Grigoriadis et al., 2013; Grote et al., 2010; Marcus, 2009). Overall, women affected by antenatal depression appear to have a higher risk of preterm birth and low birth weight compared with healthy controls, while associations with intrauterine growth restriction are less consistently observed (Grote et al., 2010). Depressive symptoms during pregnancy have also been associated with lower Apgar scores and an increased risk of pregnancy complications, with the magnitude of these associations varying according to study design, depression assessment methods, and socioeconomic context (Fekadu Dadi et al., 2020).

Several biological mechanisms have been proposed to explain the association between antenatal depression and adverse birth outcomes. Maternal depression and chronic stress are associated with dysregulation of the hypothalamic–pituitary–adrenal (HPA) axis, resulting in increased cortisol levels that may impair placental function and restrict fetal growth (Lewis et al., 2016). Moreover, elevated cortisol may also impair insulin signaling, leading to insulin resistance and hyperglycemia, representing a possible explanation of the increased risk of gestational diabetes in women with depression (Habib et al., 2022; Du et al., 2025). Other possible mechanisms include increased placental corticotropin-releasing hormone (CRH), elevated inflammatory markers such as C-reactive protein, interleukin-6, tumor necrosis factor-α, and alterations in immune function, which have also been implicated in the pathophysiology of preterm birth and low birth weight. In addition, antenatal depression may increase susceptibility to infections, such as bacterial vaginosis, which are themselves risk factors for preterm delivery (Couret et al., 2009; Marcus and Heringhausen, 2009; Orr et al., 2007; Reynolds, 2013).

From a socio-environmental perspective, antenatal depression may negatively affect maternal health-seeking behaviors and adherence to medical and psychological interventions, thereby further contributing to poor obstetric outcomes. Moreover, depression during pregnancy has been associated with behavioral risk factors known to compromise pregnancy outcomes, including smoking and substance use (Ellard et al., 1996; Kelly et al., 2002; Kyrklund-Blomberg and Cnattingius, 1998). Beyond its effects on pregnancy outcomes, prospective evidence suggests that antenatal MDD may also influence early offspring neurodevelopment. Indeed, maternal depressive symptoms have been associated with altered brain structure in their children, including amygdala hyperresponsivity (Lebel et al., 2016; van der Knaap et al., 2018). Moreover, in the PRAM-D study, maternal depression during pregnancy was associated with shorter gestational age at delivery, altered neonatal neurobehavior, and increased cortisol stress reactivity in offspring at 12 months, suggesting that antenatal depression may exert persistent effects on infant stress regulation and early neurodevelopment (Osborne et al., 2018). Consistently, a meta-analysis reported that maternal prenatal stress is associated with altered socioemotional development and behavioral difficulties in offspring (Madigan et al., 2018).

Available treatments for depressive disorders include psychotherapeutic interventions and antidepressant medications. However, a recent meta-analysis found no randomized controlled trials conducted to prospectively assess the efficacy of antidepressant medication in treating depressed pregnant women and their subsequent impact, highlighting a major gap in the current evidence base. Nonetheless, the authors confirmed the effectiveness of a broad range of psychological and non-psychological interventions for antenatal depression (Laijawala et al., 2026). According to a previous meta-analysis, antidepressants use during pregnancy has been associated with low Apgar score and preterm birth. However, it remains not fully understood the impact of the underlying disease, since authors reported increased risk also in women without antidepressants use (Vlenterie et al., 2021). Nevertheless, untreated antenatal depression has been independently associated with adverse pregnancy outcomes. Meta-analytic evidence indicates that pregnant women with untreated depression have a higher risk of preterm birth and small infant size compared with women without depression, with a trend toward greater risk among those with more severe depressive symptoms (Jarde et al., 2016). These findings underscore the clinical challenge of balancing the risks associated with pharmacological treatment against those related to untreated maternal depression.

Given its substantial burden, several professional organizations recommend routine screening for depressive symptoms during pregnancy. Available interventions include both psychotherapeutic and pharmacological treatments, highlighting the importance of individualized risk–benefit assessment and integrated perinatal mental health care ("Screening and Diagnosis of Mental Health Conditions during Pregnancy and Postpartum", 2023; Vigod et al., 2025).

### 3.2 Anxiety disorders

Anxiety disorders are among the most common mental health conditions, and they affect women more frequently than men (Farhane-Medina et al., 2022). They are characterized by the presence of excessive fear and anxiety and related behavioral disturbances (APA, 2022). According to Dennis and co-authors (2017), the prevalence of any anxiety disorder during pregnancy is 18% during the first trimester, and 15% across the second and third one (Dennis et al., 2017). Moreover, it has been reported that approximately 20.7% of women in the perinatal period (pregnancy and postpartum) meet diagnostic criteria for at least one anxiety disorder, while 5.5% meet the criteria for at least two of them (Fawcett et al., 2019). Considering panic disorder, Cantwell (2021) reported that it is more common in pregnancy than in other periods of life (Cantwell, 2021). According to Furtado and co-authors (2018), the perinatal anxiety disorder onset risk factors may be grouped into sociodemographic, psychological, biological, and obstetric and pregnancy-related factors. Particularly, a lower level of education, living with extended family members, family history of OCD, comorbid sleep disorder, prenatal oxytocin exposure, difficult delivery, and hyperemesis gravidarum are risk factors associated with perinatal anxiety onset (Furtado et al., 2018). The same authors reported also that previous negative obstetric outcomes, comorbid psychiatric diagnosis, and prenatal oxytocin exposure are risk factors associated with the perinatal worsening of anxiety symptoms (Furtado et al., 2018). Interestingly, 20 to 100% of women who were affected by obstetrical complications had higher levels of state anxiety, and it was also influenced by complication type and severity (Fischbein et al., 2019).

Considering the negative obstetric and neonatal outcomes associated with anxiety disorders during pregnancy, two different meta-analyses have highlighted that women affected by antenatal anxiety had a higher risk of preterm birth than healthy controls, whereas their offspring had a higher probability to have lower birth weight (Ding et al., 2014; Grigoriadis et al., 2018). Moreover, Grigoriadis and colleagues (2018), highlights also that offspring born from women with antenatal anxiety had a higher risk of being small for gestational age and having a smaller head circumference than controls (Grigoriadis et al., 2018). Furthermore, an original article observed that pregnant women with an anxiety disorder had a higher risk to show complications such as hypertension, diabetes mellitus, anemia, premature rupture of membranes, preterm labor, and post-partum hemorrhage. Moreover, antenatal anxiety was associated also with labor mechanical induction, cesarean section, and epidural anesthesia (Pavlov et al., 2014). Interestingly, similar findings were observed also when focusing only on panic disorder; indeed, a more recent meta-analysis focused on it underlined that panic disorder was associated with an increased risk of obstetrical and neonatal complications, such as hypertension, anemia, polyhydramnios, and low birth weight (Verhees et al., 2025).

Despite the high prevalence of perinatal anxiety disorders, no formally recognized perinatal anxiety disorder currently exists (Vigod et al., 2025). However, given the high burden of this condition during pregnancy, the American College of Obstericians and Gynecologist (ACOG) recommend that anxiety symptoms be screened at the initial prenatal visit ("Screening and Diagnosis of Mental Health Conditions during Pregnancy and Postpartum", 2023). In spite of that, currently there is no specific evidence on pharmacological treatment for perinatal anxiety; indeed, the CANMAT recommend using SSRI medications (e.g., escitalopram, sertraline) which are safer in pregnancy and lactation, although the possible negative impact of antidepressants mentioned in the previous section (Vigod et al., 2025). Stronger evidence exists for psychological treatment for perinatal anxiety disorders, with a positive impact of mindfulness programs (Pan et al., 2023; Vigod et al., 2025). Furthermore, other non-pharmacological treatments showed good efficacy in reducing anxiety symptoms during pregnancy, as for yoga, music therapy, cognitive-behavioral therapy, and the massage performed by the partner (Domínguez-Solís et al., 2021).

### 3.3 Schizophrenia spectrum disorders

Schizophrenia (SZ) affects approximately 2-3% of the population and its onset typically occurs during late adolescence or early adulthood. However, in females, onset more commonly occurs between 25 and 35 years of age, placing many women within their reproductive years at increased risk (Chan et al., 2025; B. Kirkpatrick et al., 2014). Historically, women with SZ exhibited lower fertility rates than healthy women (Vigod et al., 2012). However, early diagnosis, specialized psychosis management services, and the use of second-generation antipsychotics, have resulted in nearly comparable motherhood rates between women with SZ and healthy controls (Gentile and Fusco, 2019; Gupta et al., 2019). Nonetheless, due to their psychiatric condition and the potential impact of pharmacological treatment on maternal and fetal health, pregnant women with SZ remain at elevated risk for a wide range of adverse pregnancy and postpartum outcomes. Recent studies and systematic reviews highlight that SZ results from a complex interaction between genetic and environmental factors. Among environmental contributors, obstetric complications and perinatal events appear to increase the risk of psychosis, including the potential association of the delivery process itself with onset or exacerbation of psychotic symptoms, which is linked to increased hospitalization risk within the first postpartum month. Rates of unplanned pregnancies, miscarriages, fetal adverse outcomes, and obstetric complications are higher in these women compared to healthy mothers (Safont et al., 2025). Systematic reviews and meta-analyses of pregnant women with SZ report significantly increased risks of obstetric and neonatal complications compared to healthy controls, including: cesarean section, labor induction, both small and large for gestational age infants, gestational diabetes mellitus, preterm birth, low birth weight, premature rupture of membranes, low Apgar score, congenital malformations, antepartum hemorrhage, preeclampsia/eclampsia, postpartum hemorrhage, gestational hypertension, placental abruption, placenta previa, thromboembolic disease, and neonatal mortality (Fabre et al., 2021; Simoila et al., 2020; Tang et al., 2023). Women with SZ frequently continue antipsychotic treatment during pregnancy to prevent psychiatric relapse, particularly in severe cases. Studies indicate that prenatal exposure to first- and second-generation antipsychotics is associated with increased risk of labor induction, cesarean delivery, and neonatal special care or intensive care unit admission in treated SZ mothers compared to untreated SZ mothers (Chan et al., 2025). However, many associations become nonsignificant after adjusting for illness severity, comorbidities, and lifestyle factors (smoking, alcohol, access to prenatal care), suggesting that observed risks may be more attributable to the underlying disorder than to the medication itself (Chan et al., 2025; Forte et al., 2024).

No studies have clearly separated the effects of maternal mental illness from those of antipsychotic treatment on pregnancy, fetal, and neonatal outcomes. Evidence from one study comparing psychotropic monotherapy to untreated women with severe mental disorders showed an initial twofold increase in special care nursery admission, which became nonsignificant after adjusting for lifestyle, substance use, pre-eclampsia, and gestational diabetes (Gentile and Fusco, 2019). Future studies on the reproductive safety of individual antipsychotics are needed to guide clinical decisions on continuation, discontinuation, or switching of treatment before and during pregnancy in this vulnerable population (Chan et al., 2025).

### 3.4 Bipolar and related disorders

BDs are severe affective mood disorders characterized by recurrent mood episodes ranging from depressive to hypomanic, manic, or mixed states (American Psychiatric Association (APA), 2022). BD represents one of the leading causes of disability, morbidity, and mortality worldwide, and it is associated with substantial individual suffering, high societal costs related to health care utilization and work disability, and a markedly elevated risk of suicide, estimated to be 20–30 times higher than in the general population (Anyayo et al., 2021; Ekman et al., 2013; Pompili et al., 2013). The lifetime prevalence of bipolar I and II disorders is estimated at approximately 1–2.4% globally (Merikangas et al., 2007, 2011). Although BD affects women and men at similar rates, women tend to experience higher rates of bipolar II disorder and rapid-cycling course compared to men (Arnold, 2003; Sit, 2004). The typical age at onset ranges between 18 and 30 years, with a mean onset in the mid-twenties, overlapping with the peak reproductive years for women (Kessler et al., 2005; Sit, 2004).

Pregnancy, childbirth, and the early postpartum period, therefore, coincide with a phase of heightened vulnerability for women with bipolar disorder. Mood instability during pregnancy is common, with studies reporting that approximately 40–50% of women with bipolar I or II disorder experience a mood episode during pregnancy or the postpartum period (Di Florio et al., 2013). Moreover, hormonal fluctuations, sleep disruption, and psychosocial stressors introduced by pregnancy may further exacerbate bipolar symptoms, increasing the risk of both maternal and fetal complications. Indeed, women with BD may face a complex range of challenges during pregnancy, including illness relapse, sexually risky behaviors during manic episodes, unplanned or undesired pregnancies, and difficult decisions regarding continuation or discontinuation of psychotropic medications. As a result, pregnant women with BD appear to be at increased risk of adverse obstetric and neonatal outcomes, including gestational diabetes, gestational hypertension, antepartum hemorrhage, and hypertensive disorders such as preeclampsia or eclampsia (Etchecopar-Etchart et al., 2025; Greco et al., 2024; Mohamed et al., 2023; Tsujimoto et al., 2022). These complications may contribute to higher rates of preterm birth, low birth weight, threatened preterm labor, instrumental delivery, and cesarean section (Frayne et al., 2019; Heun-Johnson et al., 2019; Hoffman et al., 2020; MacCabe et al., 2007). Adverse neonatal outcomes more frequently reported among offspring of women with BD include prematurity, small-for-gestational-age status, neonatal intensive care unit admission, microcephaly, and congenital malformations, particularly involving the central nervous system, associating with longer hospital stays and increased health care costs (Raina et al., 2021; Rusner et al., 2016).

The mechanisms underlying these associations are likely multifactorial. From a biological perspective, bipolar disorder is characterized by dysregulation of neuroregulatory systems, including alterations in brain-derived neurotrophic factor (BDNF), chronic inflammation, oxidative stress, and impaired compensatory mechanisms over time. Those alterations may result in placental hypoperfusion, altered fetal stress responses, and impaired fetal growth (Mei-Dan et al., 2015). Moreover, elevated inflammatory markers may further contribute to placental dysfunction and an increased risk of preterm delivery (da Costa et al., 2016).

Indirect mechanisms are also likely to play a substantial role. Women with bipolar disorder are more frequently exposed to socioeconomic disadvantage and engage in health risk behaviors, including smoking, alcohol use, substance misuse, poor diet, obesity, and reduced engagement with prenatal care, all of which may further increase obstetric and neonatal risk (Sit et al., 2014).

In addition, also the pharmacological management of bipolar disorder represents a major clinical challenge during pregnancy. Lithium and atypical antipsychotics are commonly prescribed to reduce relapse risk, prolong euthymic periods, and improve overall functioning (Gentile, 2012). Regarding safety considerations, as reported in the previous section, most atypical antipsychotics are considered relatively safe, whereas lithium exposure is associated with a low risk of congenital malformations, especially during the first trimester and if administered at higher doses (Fornaro et al., 2020; Singh and Deep, 2022). Importantly, treatment discontinuation is associated with high risk of relapse during the perinatal period (Alcantarilla et al., 2023).

Overall, women with bipolar disorder are at increased risk of obstetric and neonatal complications compared with women without bipolar disorder. Discontinuation of maintenance treatment during pregnancy is associated with a high risk of relapse, whereas continued pharmacological treatment carries potential fetal risks. These challenges encountered during pregnancy and the neonatal period highlight the importance of individualized risk–benefit assessment, close monitoring, and multidisciplinary care that prioritize maternal psychiatric stability, obstetric surveillance, and neonatal well-being. Pregnant women with bipolar disorder should receive preconception counseling when possible and be closely monitored during pregnancy and the postpartum period, particularly for gestational diabetes, pregnancy-induced hypertension, and mood destabilization, in order to minimize adverse outcomes and improve long-term maternal and child health (Singh and Deep, 2022).

### 3.5 Obsessive-compulsive and related disorders

Obsessive-compulsive disorder (OCD) is a chronic psychiatric condition characterized by the presence of intrusive thoughts (obsessions), repetitive behaviors or mental actions (compulsions), or both (APA, 2022). OCD global prevalence is estimated to range between 2 and 3% and it is associated with a poorer quality of life compared to the general population (Cervin, 2023; Stein et al., 2019). Furthermore, a significant proportion of patients with OCD fail to show a full response, even after the administration of approved interventions (Martiadis et al., 2025). Considering the perinatal period, it represents a time of increased vulnerability for the OCD onset and for the exacerbation of previous conditions, as reported in the review of Hudepohl and colleagues (2022). Particularly, rates of OCD onset in pregnancy range between 2 and 22%, whereas a percentage of women with OCD ranging between 8 and 70% will experience an exacerbation during the perinatal period (Hudepohl et al., 2022). Perinatal OCD symptoms often center on the infant and present differences in the prepartum and postpartum periods. Specifically, during the pregnancy period contamination obsessions are more common, and they are usually accompanied by cleaning or washing compulsions. Conversely, postpartum onset obsessions are centered on infant harm and usually they are associated with checking or avoiding compulsions (Hudepohl et al., 2022).

Considering other conditions within the OCD spectrum, fewer data are available. However, a recent work of Grant and colleagues (2026) showed that trajectories of trichotillomania and skin picking disorder in pregnant women may differ across individuals. Moreover, they observed that women with skin picking disorder had a lower probability of receiving pharmacological treatment compared to subjects with trichotillomania. Interestingly, authors also reported a stronger correlation between skin picking disorder and postpartum depression than the one observed between this condition and trichotillomania, highlighting the importance of adequate treatment during pregnancy (Grant et al., 2026).

Regarding the etiology of perinatal OCD, evidence is still lacking. However, as for other perinatal conditions, both psychosocial and biological factors may be responsible. Particularly, the immune system may play a contributory role in the etiology of perinatal OCD, together with sleep deprivation, and serotonin, glutamate and GABA dysregulation (Hudepohl et al., 2022). Considering the obstetric history, Hudepohl and colleagues (2022) reported that primiparity represents a risk factor combining biological influences and psychosocial factors related to the impact of new responsibilities. However, in a more recent study authors did not observe the same correlation. On the contrary, they reported higher levels of OCD symptoms bidirectionally associated with preterm birth (Nakić Radoš et al., 2025). Despite some authors having investigated the association between perinatal OCD and adverse obstetrical and neonatal outcomes, data are still heterogeneous. However, some adverse conditions have been reported to be associated with OCD during pregnancy. Particularly, in a recent systematic review and meta-analysis authors underlined that maternal OCD during pregnancy may negatively impact both the mother and the fetus. Particularly, it may be associated with an increased risk of pre-eclampsia, placental abruption, cesarean section, postpartum hemorrhage, preterm birth, low birth weight, low Apgar score at 5 min, neonatal hypoglycemia, neonatal respiratory distress, and major congenital malformation (Aujla et al., 2024). Interestingly, in one observational study authors reported that by correcting for other psychiatric comorbidities the increased risks of obstetrical and neonatal complications were attenuated but not eliminated, suggesting a partially independent contribution of OCD-related pathology (Fernández de la Cruz et al., 2023). However, most evidence derives from large population-based observational studies, and causal mechanisms remain to be fully elucidated.

Given the burden of perinatal OCD in the maternal wellbeing, impacting maternal and family functioning as well as obstetrical and neonatal outcomes, it is important to recognize and treat this condition. However, women with perinatal OCD may experience several barriers related to diagnosis and treatment, as reported by Burton and colleagues (2022). Particularly, women may feel worried about disclosing their symptoms to maternity care professionals and might be concerned about the opinion of their family members or of the wider society. Moreover, women may be afraid of their baby being taken away if they disclose the nature of their obsessions. Nevertheless, women with OCD symptoms may show reluctance in taking medications due to the unknown effects on the fetus, whereas those who are already under treatment for OCD may stop the medications for the same reasons (Burton et al., 2022). However, according to CANMAT guidelines there are no specific pharmacological treatments studied for pregnancy and lactation, and the available treatment should be used with caution (Vigod et al., 2025). Moreover, pharmacokinetics changes in pregnancy should be taken into account when a pharmacological treatment is prescribed; indeed, plasma concentration may drop by 40 to 50% by the end of pregnancy (Hudepohl et al., 2022). Regarding psychological intervention and neuromodulation techniques such as rTMS, they showed a higher quality of evidence (Vigod et al., 2025).

### 3.6 Trauma- and stressor-related disorders

PTSD is a pathological reaction to one or more traumatic events. This exposure leads to symptoms including re-experiencing the event, avoidance of associated stimuli, and negative alterations in cognition, mood, and arousal (APA, 2022). Although PTSD – regardless of its onset – is a concern at any time, the presence of these symptoms during the perinatal period can lead to additional challenges, as it may increase the risk of gestational complications and adverse birth outcomes (McKenzie-McHarg et al., 2015). Women may experience PTSD during pregnancy either as a continuation of pre-existing PTSD related to earlier life events, or because of trauma encountered during gestation, such as labor complications, emergency delivery, intimate partner violence, or other pregnancy-related traumatic experiences. On average, the prevalence of PTSD during pregnancy is 3.3% in the general population, with an additional 4% prevalence in the postpartum period, mostly related to traumatic events occurring during childbirth (Yildiz et al., 2017). Rates increase to 15–18% among high-risk women who experience severe complications during pregnancy or childbirth, preterm delivery, intrauterine fetal death, or emergency cesarean section (Horsch et al., 2015, 2016, 2017). Some studies suggest that prenatal PTSD is associated with pregnancy complications such as ectopic pregnancy, spontaneous abortion, hyperemesis, preterm contractions, and excessive fetal growth (Lutgendorf et al., 2022; Seng et al., 2001). In meta-analysis including sixteen studies with 4118 participants with prenatal PTSD, maternal prenatal PTSD was associated with an increased risk of low birth weight, preterm birth, and reduced gestational age (Sanjuan et al., 2021). However, a more recent meta-analysis has reported conflicting results. Zitoun et al. (2025), considering 40 studies including 11,750 pregnant participants with PTSD, found that some studies demonstrated a significant association between maternal PTSD and low birth weight, lower gestational age, and preterm birth, while others did not. They also examined the association between prenatal PTSD and neonatal head circumference, highlighting some degree of association between prenatal PTSD and reduced neonatal head circumference (Zitoun et al., 2025). Other studies conducted on pregnant veteran women identified a significant association between maternal PTSD and an increased risk of gestational diabetes mellitus, hypertension, and preterm birth (Katon et al., 2014; Shaw et al., 2017). Prenatal PTSD is also associated with high rates of psychiatric comorbidities, including anxiety, depression, and substance use, which may further increase the risk of preterm birth, low birth weight, and impaired fetal growth (Morland et al., 2007). The described obstetric complications may be linked to sustained activation of the maternal hypothalamic-pituitary-adrenal (HPA) axis in response to chronic psychological stress, resulting in neuroendocrine abnormalities, including a suppressed cortisol response in both mother and fetus, and alterations of the intrauterine environment during pregnancy (Bowers and Yehuda, 2016; Shapiro et al., 2013). As already reported, altered circadian cortisol profiles have been associated with prenatal psychological distress, depression, anxiety, preterm birth, and fetal weight (Sanjuan et al., 2021). Specifically, in relation to prenatal PTSD, chronic stress associated with PTSD may lead to hormonal dysregulation, including altered vasopressin and oxytocin levels and increased cortisol, which can directly predispose women to obstetric complications and negatively impact fetal growth and development (Friedman and McEwen, 2004; R. D. Marshall and Garakani, 2002). PTSD has also been linked to immune system dysregulation, potentially contributing to inflammatory processes and other pregnancy complications (Zitoun et al., 2025). Rinne et al. (2024) reported that preconception maternal PTSD symptoms and second-trimester inflammation were associated with shorter telomere length in offspring during early childhood. These findings suggest that intergenerational transmission of parental trauma may occur, at least in part, through accelerated biological aging processes, providing further evidence that prenatal pro-inflammatory processes can influence telomere length in the child (Rinne et al., 2024). In conclusion, multiple studies suggest that prenatal PTSD can pose direct risks to maternal health and prenatal fetal bonding, but its impact on birth outcomes and fetal development remains unclear, and the literature shows inconsistent results (Cook et al., 2018; Grigoriadis et al., 2018; Zitoun et al., 2025). Evidence indicates that PTSD may be associated with low birth weight and reduced gestational age, similar to prenatal maternal depression (Sanjuan et al., 2021). However, existing studies are not yet exhaustive and primarily focus on the relationship between PTSD and the postpartum period, as well as mother-infant interactions.

### 3.7 Borderline personality disorder

Borderline Personality Disorder (BPD) is characterized by instability of interpersonal relationships, affect, and self-image, together with marked impulsivity. Despite an early onset of BPD being described, the symptoms usually start in early adulthood. Therefore, BPD affects women of reproductive age with potential consequences on offspring development (APA, 2022; Nguyen et al., 2024). The prevalence of BPD in the general population is estimated at 2.7%, whereas meta-analytic evidence suggests a pooled prevalence of approximately 14% among clinical perinatal samples (Prasad et al., 2022).

BPD in the perinatal period is associated with several obstetric and neonatal complications, such as the increased risk of threatened preterm labor, urinary tract infections, pre-eclampsia, antepartum hemorrhage, and premature rupture of membranes (Nguyen et al., 2024). Moreover, a retrospective study underlined that perinatal BPD was associated with an increased risk of referral to child protective services or parenting support services after delivery, together with the increased risk of low birth weight and low Apgar scores, preterm birth, and the need for resuscitation and referral to a special care nursery or neonatal intensive care unit (Blankley et al., 2015). Furthermore, Pare-Miron and colleagues (2016), in a retrospective study on a large cohort, interestingly divided the obstetrical outcomes into intrapartum complications, delivery complications, and postpartum complications. In the first group, they observed an increased risk of gestational diabetes, premature rupture of membranes, and chorioamnionitis in women with BPD, whereas cesarean delivery was more frequent in women with BPD compared to controls. Focusing on postpartum and neonatal complications, women with BPD had a higher risk of venous thromboembolism and preterm birth (Pare-Miron et al., 2016). Interestingly, a meta-analysis investigating the relationship between personality disorders in general and early birth outcomes highlighted that the risk of low birth weight and Apgar scores, and preterm birth are higher in personality disorders compared to the general population, although two out of five studies focused on BPD (C. A. Marshall et al., 2020). However, most available evidence derives from retrospective and observational studies, and the independent contribution of BPD relative to psychiatric comorbidities remains to be fully clarified.

Despite the negative impact of BPD on both mother and baby, the literature on clinical management is sparse. Accordingly, a recent review proposed two different treatment approaches such as the inpatient mother-baby unit protocols and the specialized group therapy (Sved Williams and Hill, 2023). Moreover, home-visiting interventions and child-parent psychotherapy have also been studied as treatments for BPD in the perinatal period (May et al., 2023). However, dialectical behavior therapy (DBT) is the most studied treatment approach for BPD in the perinatal period, focusing in some cases on perinatal adaptation or mother-infant protocols (Sved Williams and Hill, 2023). Given that personality disorders are associated with poor engagement with maternity services and perinatal mental health services in addition to negative obstetrical and neonatal outcomes, according to the National Institute for Health and Care Excellence (NICE) guidance, recommendations for research in antenatal and postnatal mental health should include psychological interventions focused on the mother-baby relationship and structured clinical management for treating moderate to severe personality disorders (National Institute for Health and Care Excellence, 2020).

Across diagnostic categories, several recurring sources of heterogeneity emerge that may contribute to inconsistencies in reported obstetric and neonatal outcomes. As observed in studies on schizophrenia-spectrum disorders and OCD, adjustment for illness severity, psychiatric comorbidities, and metabolic or lifestyle factors often attenuates risk estimates, suggesting a substantial role of confounding (Chan et al., 2025; Fernández de la Cruz et al., 2023). Moreover, variability in diagnostic ascertainment, outcome definitions, pharmacological exposure, and socioeconomic context further complicates causal interpretation. Recognizing these shared methodological challenges is essential for a balanced interpretation of existing evidence and for the design of future prospective and longitudinal research.

## 4 Common pathophysiological mechanisms and shared risk factors in perinatal psychiatry

**Fig. 1:**
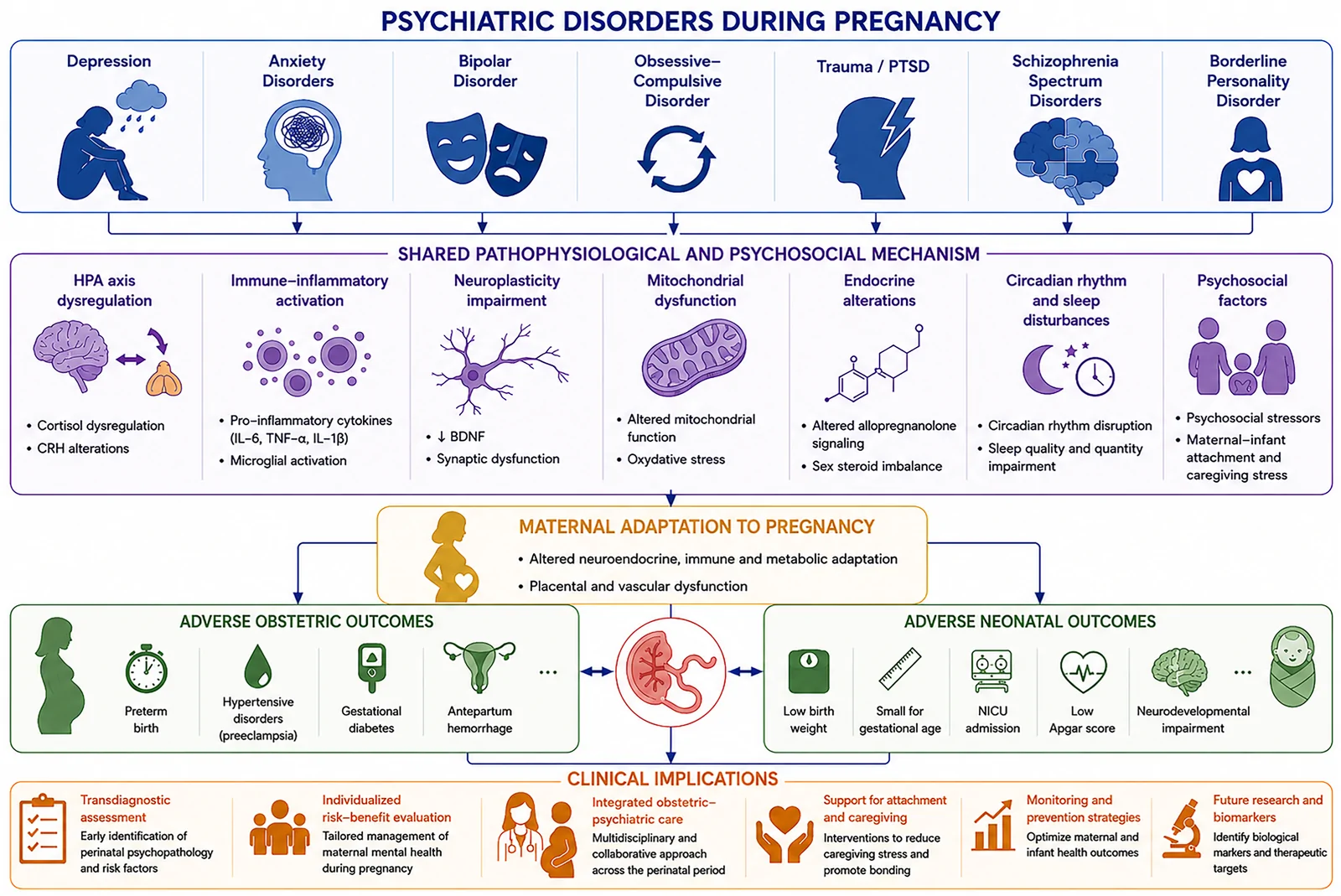
Proposed transdiagnostic framework linking psychiatric disorders during pregnancy to adverse obstetric and neonatal outcomes. Major psychiatric disorders during pregnancy – including mood, anxiety, psychotic, obsessive-compulsive, trauma-related disorders, and borderline personality disorder – may converge on shared biological and psychosocial mechanisms, including hypothalamic–pituitary–adrenal (HPA) axis dysregulation, immune-inflammatory activation, impaired neuroplasticity, mitochondrial dysfunction, endocrine alterations, circadian rhythm and sleep disturbances, and psychosocial factors. These mechanisms may contribute to altered maternal adaptation to pregnancy, ultimately increasing the risk of adverse obstetric and neonatal outcomes. The figure also highlights the potential clinical implications of a transdiagnostic approach, including early identification, individualized risk–benefit assessment, integrated obstetric–psychiatric care, and future research aimed at improving maternal and offspring outcomes.

Perinatal psychiatry is increasingly conceptualized as an interdisciplinary field at the intersection of neurobiology, public health, and psychosocial determinants, where vulnerability to mental disorders arises from the dynamic interaction between biological and environmental factors (Pinna and Pathak, 2025). Fig. 1 summarizes the proposed transdiagnostic framework linking psychiatric disorders during pregnancy, shared biological and psychosocial mechanisms, and adverse obstetric and neonatal outcomes.

From a neurobiological perspective, dysregulation of the hypothalamic–pituitary–adrenal (HPA) axis and activation of pro-inflammatory immune pathways represent two of the most consistently implicated mechanisms in perinatal affective disorders, particularly depression (Silva-Fernandes et al., 2024; Sun et al., 2026⁠). Hyperactivation of stress-response systems during pregnancy may lead to sustained cortisol secretion, which has been associated not only with perinatal depression but also with bipolar spectrum disorders, potentially explaining the partial overlap in obstetric and neonatal outcomes observed across diagnostic categories.

In parallel, increasing evidence supports a role for neuroinflammatory processes in disrupting neuroplasticity pathways. Elevated peripheral cytokines, including IL-6 and TNF-α, have been associated with perinatal depressive symptomatology, and are thought to interfere with intracellular signaling cascades such as the mammalian target of rapamycin (mTOR) pathway, ultimately contributing to reduced expression of neuroplastic mediators such as brain-derived neurotrophic factor (BDNF) and impaired synaptic plasticity (Bertollo et al., 2025; Jafarabady et al., 2024; Silva-Fernandes et al., 2024). Notably, reduced maternal and fetal BDNF levels have been reported in association with perinatal depression and anxiety-related conditions, suggesting potential intergenerational effects on neurodevelopmental outcomes (Uguz et al., 2013; Jafarabady et al., 2024). Hormonal mechanisms further interact with these inflammatory pathways, particularly through fluctuations in reproductive steroids such as estrogen and progesterone and their neuroactive metabolites. In this context, altered allopregnanolone signaling has been strongly implicated in peripartum mood disorders, and pharmacological modulation of this pathway via brexanolone and zuranolone has demonstrated clinical efficacy in postpartum depression (Standeven et al., 2022; Barnes et al., 2024)

Beyond endocrine and immune alterations, emerging evidence highlights the importance of circadian rhythm and sleep–wake disruption as a shared mechanistic pathway across perinatal psychiatric conditions. Sleep fragmentation and circadian misalignment are highly prevalent during pregnancy and the postpartum period and have been prospectively associated with increased risk of both depressive and anxiety symptoms, likely via effects on emotional regulation and stress responsivity (Cox, 2025). In addition, genetic susceptibility and epigenetic modifications—particularly those involving estrogen receptor signaling and stress-related gene regulation—appear to contribute to inter-individual variability in risk, supporting a model of gene–environment interaction in perinatal vulnerability (Zorzini and Ehlert, 2025).

More recently, mitochondrial dysfunction and oxidative stress have been proposed as additional convergent mechanisms linking metabolic dysregulation with inflammatory and neuroendocrine abnormalities in mood disorders, including during the perinatal period (Ceylan et al., 2024). These biological vulnerabilities are further modulated by psychosocial determinants such as prior psychiatric history, exposure to trauma or intimate partner violence, socioeconomic disadvantage, chronic medical conditions, and limited access to mental health care, all of which consistently increase the risk for perinatal depression and related disorders (Bränn et al., 2024; Hudepohl et al., 2022).

Finally, relational and developmental processes, particularly mother–infant bonding and caregiving stress, represent important downstream pathways through which perinatal psychopathology may exert intergenerational effects. Mother-infant bonding refers primarily to the mother's emotional connection to the infant, whereas interaction concerns observable dyadic behaviors such as sensitivity, responsiveness, and synchrony; parental mentalization or reflective functioning refers to the capacity to understand the infant's behavior in terms of underlying mental states (Tichelman et al., 2019; Vilaseca et al., 2025; Rutherford et al., 2017). Disturbances in bonding and caregiving sensitivity can further amplify maternal stress responses, reinforcing dysregulation across neuroendocrine and immune systems in a bidirectional feedback loop that impacts both maternal mental health and infant neurodevelopment. Consistent with this framework, systematic evidence indicates an association between maternal psychological distress and difficulties in mother–infant bonding, with depression and anxiety representing the most extensively investigated conditions (Tichelman et al., 2019; O'Dea et al., 2023). Evidence on these relational processes across psychiatric conditions other than depression and anxiety remains more limited, although available findings suggest impaired mother–infant interaction in severe mental disorders, particularly psychotic and bipolar disorders, highlighting the need for longitudinal, transdiagnostic research (Vilaseca et al., 2025).

Overall, the convergence of these interconnected mechanisms supports a transdiagnostic biopsychosocial framework of perinatal psychiatric vulnerability, providing a plausible explanation for the shared obstetric and neonatal outcomes observed across diagnostic categories and underscoring the need for integrated prevention and intervention strategies in perinatal mental health care.

## 5 Future perspectives

Given the narrative design of this review, findings should be interpreted considering the heterogeneity of available evidence and the absence of quantitative synthesis. Nevertheless, several consistent patterns and key research gaps can be identified across diagnostic categories.

Despite increasing recognition of perinatal mental health as a public health priority, evidence on the impact of psychiatric disorders on obstetric and neonatal outcomes remains fragmented and methodologically heterogeneous (Howard and Khalifeh, 2020). Several conditions remain underinvestigated, contributing to inconsistencies in the interpretation of findings (Vigod et al., 2025). Moreover, it remains unclear whether the different conditions are associated to different risk profiles based on specific biological and psychosocial factors or they share the same associations through common pathways.

Future research should prioritize well-designed prospective and longitudinal studies capable of disentangling the independent and combined effects of different psychiatric conditions across pregnancy and the postpartum period (Howard and Khalifeh, 2020; Vigod et al., 2025). Greater attention to underinvestigated conditions may enhance current knowledge in perinatal psychiatry and clarify the impact of mental health conditions during pregnancy and the peripartum period. Moreover, the adoption of standardized obstetric and neonatal outcome measures would facilitate integration across studies and diagnostic categories. By characterizing risk profiles and clinical phenotypes, future research may allow the identification of novel biological and social determinants of disease.

## 6 Clinical implications and conclusions

The findings summarized in this review highlight the clinical relevance of considering a broad range of psychiatric disorders during the perinatal period, beyond depressive disorders alone. To our knowledge, it represents a novel integrative review grouping several psychiatric conditions and examining the associations between perinatal mental health conditions and obstetric and neonatal outcomes. Although the exact mechanisms underlying these associations remain to be fully elucidated, common neuroinflammatory and endocrine alterations have been observed in different conditions. Given the increased risk of several complications, including placental dysfunction, hypertension (including pre-eclampsia), preterm birth, small for gestational age fetuses, and low birth weight, early identification and appropriate management of psychiatric disorders may represent a key component of comprehensive perinatal care.

From a clinical perspective, integrating mental health assessment into routine obstetric care and strengthening collaboration between obstetric and mental health professionals may help address the complex needs of women during pregnancy and the postpartum period, potentially reducing the risk of adverse outcomes. Moreover, by enforcing this collaboration, women with previous mental health conditions may receive adequate counseling before pregnancy and appropriate attention during pregnancy and post-partum, improving their overall quality of life. Nonetheless, particular attention should be given to severe and underrecognized conditions, comorbid presentations, and continuity of care across the perinatal trajectory.

In conclusion, this narrative review provides a transdiagnostic overview of perinatal psychiatric disorders and their association with obstetric and neonatal outcomes. By integrating evidence across diagnostic categories, it enables the identification of shared risk patterns and highlights areas where targeted clinical strategies and future research efforts may contribute to improved maternal and offspring outcomes.

## CRediT authorship contribution statement

**Tiziano Prodi:** Writing – review & editing, Writing – original draft, Investigation, Conceptualization. **Melissa Rosa:** Writing – original draft, Investigation. **Marta Spinoni:** Writing – original draft, Investigation. **Claudio Singh Solorzano:** Writing – review & editing. **Maria Grazia Di Benedetto:** Writing – review & editing. **Marco Andrea Riva:** Supervision. **Bernardo Dell’Osso:** Supervision. **Annamaria Cattaneo:** Writing – review & editing, Supervision, Conceptualization.

## AI declaration statement

During the preparation of this manuscript, AI tools were used for language editing and grammar checking (ChatGPT, Grammarly), as well as for the generation and refinement of the graphical figure (ChatGPT). All AI-generated outputs were carefully reviewed, edited, and verified by the authors, who take full responsibility for the content of this manuscript. The use of these tools was consistent with COPE and ICMJE recommendations.

## Declaration of competing interest

Authors have no conflicts of interest to declare in relation to the content of the present manuscript.

## Data Availability

No data was used for the research described in the article.
